# Case Report: *Sarcina ventriculi*, a masquerade of motility

**DOI:** 10.3389/fgstr.2025.1607667

**Published:** 2025-08-05

**Authors:** Katherine Westbrook Cates, Catherine Hudson

**Affiliations:** Louisiana State University Health Sciences Center, New Orleans, LA, United States

**Keywords:** bacteria, motility, *Sarcina*, gastroenterology, infectious disease

## Abstract

*Sarcina ventriculi* is a rare bacterium that has the potential to cause severe disease in the gastric mucosa. According to our search, there are less than 100 prior published case reports. This case discusses a 69-year-old man who presented to the hospital with severe gastric distention noted on CT of the abdomen with intractable nausea and emesis, for which endoscopy was performed and ruled out gastric outlet obstruction, with biopsies resulting as *S. ventriculi*. This bacterium has previously been reported to cause gastric dysmotility, as well as severe side effects including gastric perforation.

## Introduction

*Sarcina ventriculi* is a rare, Gram-positive anaerobic bacterium with carbohydrate fermentation ability that is commonly found in unwashed vegetables. It was first coined in 1842 by Wilson and Goodsir after isolation from a patient with intractable emesis (1). *S. ventriculi* has been studied in limited research cases as causing a significant delay in motility with gastric outlet obstruction concerns, gastric ulcerations, gastric erythema, and even documented cases of gastric perforation. It is unknown how this bacterium causes such dysmotility, but it is theorized that it alters the normal muscularis mucosa via carbohydrate fermentation. Notably, this bacterium can survive in extremely low pH environments, such as the gastric acid in the stomach.

As of 2013, only eight cases of this rare bacterium have been published (2). In 2021, it was expressed that only 48 cases of this bacterium have been documented. Prior to the documented cases in humans, this bacterium was isolated from animal intestines and is commonly studied in veterinary medicine as an etiology for gastritis, gastric perforation, and dysmotility (3). Due to the limited number of cases and the many unknowns about the disease course of this bacterium, we present a case of isolated *S. ventriculi* in a patient with severe gastric dysmotility.

## Case report

We present a 69-year-old man with grade D esophagitis, hypertension, duodenal angioectasias, chronic obstructive pulmonary disease, pulmonary mass, and recurrent small bowel obstructions who presented to the emergency department (ED) with intractable nausea and bilious vomiting with severe abdominal pain in the epigastric region of 2 day’s duration. CT scan of the abdomen and pelvis was performed, revealing concern for gastric outlet obstruction with severe gastric distention.

At 2 months prior to presentation, the patient was hospitalized for hematemesis. Subsequent esophagogastroduodenoscopy (EGD) revealed grade D esophagitis and erosive gastropathy, which was biopsied with the pathology resulting as rare *S. ventriculi*. The patient was prescribed outpatient ciprofloxacin for 10 days, with patient-reported completion of therapy without missed doses. He returned after completion of the ciprofloxacin treatment with recurrent nausea with vomiting and abdominal pain.

**Figure 1:**
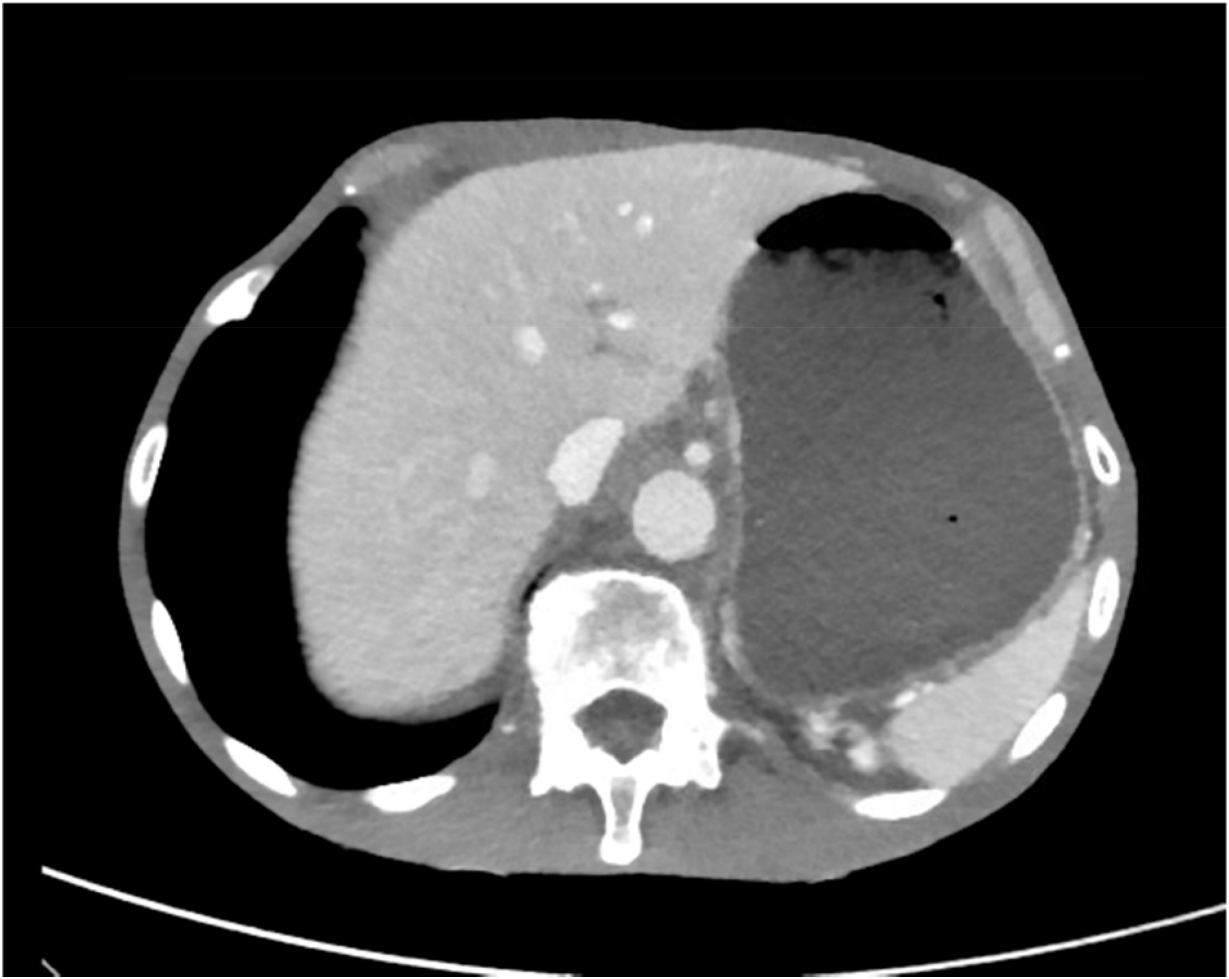
CT imaging of gastric distention.

**Figure 2:**
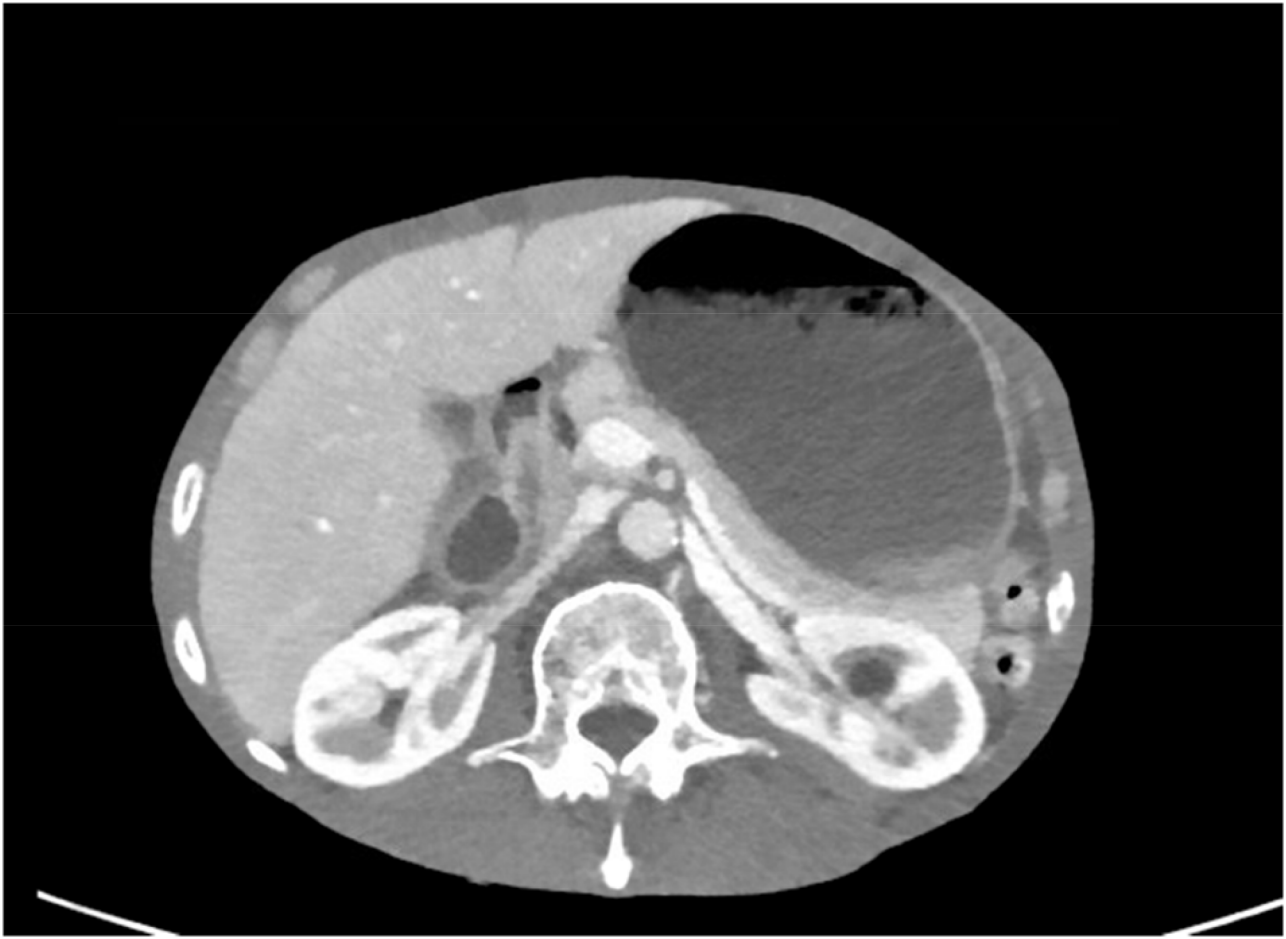
CT imaging of gastric distention.

On the CT scan showing gastric outlet obstruction, there was a significant amount of food noted on the radiographic images (**Figures 1**, **2**), for which a nasogastric tube was placed and intermittent wall suction started. This produced significant output over a span of 3 days, in which his symptoms of nausea and vomiting improved. The EGD performed following nasogastric tube decompression showed food residue and no evidence of gastric outlet obstruction, making dysmotility the most likely etiology. Repeat biopsies were taken during endoscopy, which were negative for *S. ventriculi* for a second time. Decision to empirically treat with ciprofloxacin plus metronidazole combination therapy was deferred as the pathology was negative on repeat EGD. The patient continued to clinically improve and was able to advance diet without repeat gastric distention.

## Discussion

In the limited previously published literature on *S. ventriculi*, the majority of the documented cases are consistent with similar presentations to our patient with gastric dysmotility, gastric distention, and correlating epigastric pain. After days of nasogastric tube decompression, the patient underwent endoscopy with removal of the residual food content, and gastric outlet obstruction was ruled out. This made the underlying *S. ventriculi* the likely etiology of the gastric distention and dysmotility. The mechanism by which this bacterium causes the dysmotility is unclear. It is important to note that this patient had no other comorbidities that could have led to this gastric dysmotility, such as gastroparesis or diabetes as a couple of examples. It is also pertinent that he had no offending medications after medication review to cause this presentation or dysmotility, supporting *S. ventriculi* as the inciting factor for the presentation of this patient.

It is also important to discuss that this patient’s presentation was after completion of a full course of ciprofloxacin with negative repeat biopsy. This was prescribed approximately 2 months prior to hospital presentation, and the patient reported completing the entire course of antibiotic. The utility of empirically retreating with ciprofloxacin plus metronidazole *versus* no further treatment was discussed with the infectious disease consulting physician during inpatient stay. The final decision was to hold off on therapy and re-biopsy during upcoming endoscopy. A previous documented pediatric case resulted in the complete resolution of infection after ciprofloxacin and metronidazole combination therapy, including repeat negative biopsies (4).

The negative biopsies on repeat EGD could be secondary to the completed course of ciprofloxacin or could be attributed to a false-negative biopsy result. The patient experiencing symptoms of dysmotility for several weeks after biopsy confirmed that the exposure supports the theory of the muscularis layer of the gastric mucosa being targeted as the pathogenesis of *S. ventriculi*. This could indicate that this bacterium causes lingering effects to the mucosa after the initial infection, or this may support *S. ventriculi* being more of an incidental bacterium to a secondary process. More studies are needed to support this theory, in particular those on human cases (2). In a study characterizing several cases of this bacterium in animals, the pH tolerance of the bacterium was emphasized, making another theory consistent with the bacterial overgrowth of *S. ventriculi* causing the correlating symptoms as seen in our case (5).

While this bacterium is becoming more prevalent among humans, this case is relevant to the growing knowledge of isolation, treatment, and course of disease for *S. ventriculi*. It is particularly important to understand how this bacterium causes severe disease and notably affects the gastric mucosa. This is imperative to understand the clinical course for patients with symptoms secondary to *S. ventriculi*, as well as for the recurrence of symptoms. More studies are needed to standardize the approach to care for this patient population.

## Data Availability

The original contributions presented in the study are included in the article/Supplementary Material. Further inquiries can be directed to the corresponding author.
